# Hemizygous Moesin (MSN) Gene Deletion in an Adult With Chronic Neutropenia

**DOI:** 10.1155/crii/3860726

**Published:** 2024-12-31

**Authors:** Yandy Marx Castillo-Aleman, Francisco Sotomayor-Lugo, Sherjeel Sana, Nameer Abdul Raheem Kadhum Al-Saadawi, Yendry Ventura-Carmenate, David Dennison, Gianina Statache, Julieta Osorio-Zuluaga, Ahmad Raza, David Grossman

**Affiliations:** ^1^Department of Immunology, Abu Dhabi Stem Cells Center (ADSCC), Abu Dhabi, UAE; ^2^Department of Molecular Biology, Abu Dhabi Stem Cells Center (ADSCC), Abu Dhabi, UAE; ^3^Department of Medical Oncology and Hematology, Oncology Institute, Cleveland Clinic Abu Dhabi (CCAD), Abu Dhabi, UAE; ^4^Department of Hematology, Abu Dhabi Stem Cells Center (ADSCC), Abu Dhabi, UAE; ^5^Department of Rheumatology, Abu Dhabi Stem Cells Center (ADSCC), Abu Dhabi, UAE

**Keywords:** immunodeficiency, leukopenia, moesin, *MSN* gene mutation, neutropenia, primary immunodeficiency disease

## Abstract

X-linked moesin-associated immunodeficiency (X-MAID) is a recently identified combined immunodeficiency caused by a mutation in the moesin (*MSN*) gene. It is characterized by cytopenias, hypogammaglobulinemia, poor immune response to vaccine antigens, and increased susceptibility to early-life infections. We report a patient with adult-onset neutropenia, lymphopenia, inadequate response to the pneumococcal polysaccharide vaccine (PPSV23), and recurrent bacterial infections associated with a hemizygous *MSN* deletion. Notably, the patient has no history of significant childhood infections, cytopenias, or hypogammaglobulinemia. Although only a few cases have been documented worldwide, we underscore the importance of whole-genome sequencing (WES) in diagnosing this atypical immunodeficiency disease in adulthood. Moreover, this report may shed light on our understanding of further variants of X-MAID and enrich the known spectrum of the disease.

## 1. Introduction

In 2016, Lagresle-Peyrou et al. [1] reported seven male patients who exhibited profound lymphopenia early in life, fluctuating monocytopenia and neutropenia, hypogammaglobulinemia, poor immune response to vaccine antigens, and increased susceptibility to bacterial and varicella zoster virus (VZV) infections. In this seminal paper, the authors established a causal link between an ezrin–radixin–moesin (ERM) protein mutation and a novel primary immunodeficiency, X-linked moesin-associated immunodeficiency (X-MAID). They observed hemizygous mutations in the moesin (*MSN*) gene, which codes for the MSN protein, in all analyzed patients [1].

One year later, Delmonte et al. [2] described the first case of X-MAID identified through screening for severe combined immunodeficiency (SCID) in a male who exhibited profound lymphopenia since the third week of life and showed similar features as previously reported. Similarly, in 2017, Xiao-ying et al. [3] diagnosed this immunodeficiency in an 8-year-old boy with recurrent pulmonary and intestinal infections and eczema.

In 2018, Bradshaw et al. [4] published a case of a young male proband who presented with recurrent bacterial and VZV infections in childhood and persistent lymphopenia into early adulthood. X-MAID was confirmed after remaining undiagnosed for 24 years.

Following those initial reports, Henrickson et al. [5] described three cases of X-MAID in 2019, presenting with SCID and treated with hematopoietic stem cell transplantation (HSCT) and myeloablative conditioning. In 2021, Urdinez et al. [6] also described an Argentinian family with two affected brothers, both with a history of neutropenia and childhood infections and one of them suffered from recurrent retinal detachment. Fang et al. [7] reported a 5-year-old boy with X-MAID, presenting as an inflammatory bowel disease (IBD)-like disease with no previous history of recurrent infections, except for a single episode of mild pneumonia at 4-months-old.

More recently, Li et al. [8] reported two brothers with X-MAID presented with bronchiectasis, emphysema, and repeated lung infections after 6 months of age, reaching a total number of 18 patients with this immunodeficiency reported worldwide.

Despite repeated infections in early life being a common clinical presentation of X-MAID, we report a case of adult-onset profound neutropenia with no history of significant childhood infections, cytopenias, or hypogammaglobulinemia, in which a hemizygous deletion of the *MSN* gene was detected. The clinical features were consistent with X-MAID.

## 2. Case Presentation

A 35-year-old Arab male patient presented to our hospital with chronic neutropenia, poor antibody response to the pneumococcal polysaccharide vaccine (PPSV23), and increased susceptibility to upper respiratory tract bacterial infections over the past 7–8 months. Six months prior to the onset of neutropenia, the patient had an uncomplicated parvovirus B19 infection. He also reported experiencing some B symptoms, such as night sweats and weight loss, which resolved on their own 2 years ago. There was no other significant personal or family history.

**Figure 1:**
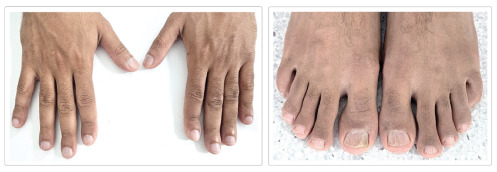
Major phenotypic features. Nail and digital clubbing with striae in both the fingernails and toenails, and clinodactyly was more pronounced in the second finger of his left hand.

Physical examination revealed no lymphadenopathies or hepatosplenomegaly. The most notable findings were Hippocratic fingers and nails, with varying degrees of clinodactyly of the distal phalanges (Figure 1). Chest X-ray, abdominal ultrasound, brain MRI, and echocardiogram were unremarkable.

**Figure 2:**
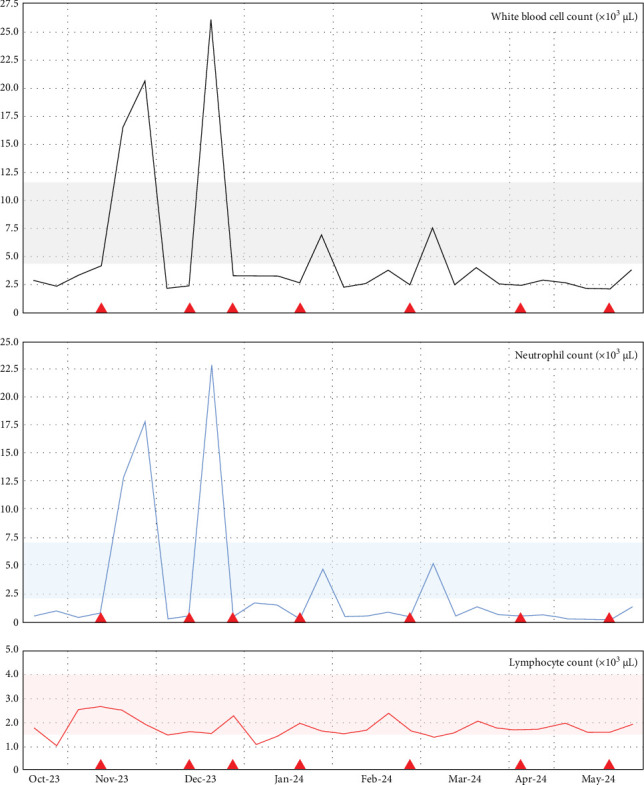
Peripheral blood-cell counts and their response to granulocyte colony-stimulating factor (G-CSF) administration. Fluctuating cytopenias and their response to G-CSF administration (red arrowheads) after onset. The timeline is expressed as MMM-YY. The highlighted gray, blue, and red areas correspond to the reference ranges for white blood cell, neutrophil, and lymphocyte counts, respectively.

His neutropenia had reached 0.25 × 10^3^ cells/µL (Grade 4), but improved with intermittent subcutaneous doses of granulocyte colony-stimulating factor (G-CSF) administered three times weekly when the neutrophil count fell below 0.75 × 10^3^ cells/µL.  Figure 2 summarizes the fluctuating cytopenias.

Compared to the immune profiling at onset, a 4-month follow-up immunological assessment showed total serum immunoglobulin (Ig) levels and quantitative B- and T-cell subsets within normal ranges, but a slight and progressive decrease in the CD4 and CD8 T cell counts with inversion of CD4/CD8 ratios (Table 1).

Peripheral blood films showed normocytic and normochromic erythrocytes, platelets, and leukocytes with normal morphology, despite severe neutropenia. Bone marrow aspirate revealed adequate granulopoiesis and maturation, while a bone marrow core biopsy indicated trilineage hematopoiesis without morphologic abnormalities, increased blasts, or abnormal B- and T-cell populations as per flow cytometry.

Whole-genome sequencing (WES) of peripheral blood did not detect mutations in the *ELANE* or *GATA2*, typically associated with cyclic neutropenia or myelodysplastic syndrome (MDS). However, a hemizygous deletion of the *MSN* gene spanning 2816 base pairs was identified. This copy number variant (CNV) was located at cytoband Xq12, with chromosome position chrX:65622070-65624886 (GRCh38.p14). Of note is that we could not study the *MSN* gene expression in his mother.

## 3. Discussion

Various short variants (single-nucleotide polymorphisms (SNPs) and indels) documented in this region in the Ensembl database (https://www.ensembl.org) involve intronic or noncoding transcript exon variants without associated phenotypes. The identified structural variant in this patient overlaps with two transcripts. The first transcript, ENST00000609672, encodes a protein, but the deletion region affects only intronic sequences. The second transcript, ENST00000609205, is an undefined protein-coding CDS, but the deletion affects intronic and noncoding transcript exon sequences as well.

Within the deletion range, reported gain/loss CNVs (regions found duplicated in some individuals—or alleles—and deleted in others) include a structural variant (nsv4051866) at chromosome position chrX:65623043-65624625 (GRCh38.p14), involving a 1583 base pairs deletion. This variant also overlaps with intronic regions of the two mentioned transcripts and noncoding transcript variants, with no associated phenotype reported. Despite evidence from the Ensembl database, the effect of intron losses on gene expression may be underestimated. Additionally, current regulatory region data is limited, with interaction maps varying across cell types [9] and tissue-specific enhancers [10] possible influencing cell type-specific gene expression regulation, thereby, complicating studies involving intronic regions.

Although intronic deletions do not affect coding sequences directly, they can influence transcription rate, splicing process, RNA stability, or a combination thereof. Intronic CNVs, which do not overlap with any annotated isoform exons, constitute 63% of all CNVs but remain the least studied [11] and are often classified as variants of uncertain significance. Significant variations in alternative transcript proportions have been observed in genes with intronic deletions. The balance between unspliced- and spliced-messenger RNA, dependent on intronic variations, is a cell type-specific signature that predicts gene expression changes in individual cells [12].

Differential diagnoses considered polyclonal disorders (related to infections, autoimmunity, and medications), clonal myeloid and lymphoid diseases (such as large granular lymphocytic leukemia and Felty syndrome), and cyclic and constitutional (benign ethnic) neutropenias. However, as Valent [13] noted, many peripheral cytopenias categorized as “idiopathic cytopenias of unknown significance” or “clonal cytopenias of unknown significance” may represent MDS prephases that precede hematopoietic neoplasms.

Since we could not confirm whether the patient's mother is affected, a sporadic gene mutation may have led to the onset of the condition in adulthood. However, the presence of chronic and profound neutropenia, transient lymphopenia, inadequate responsiveness to PPSV23, and an intronic CNV in the *MSN* suggests a plausible association with X-MAID in this patient. While X-MAID typically manifests in early life with symptoms of SCID [1, 2], the *MSN* deletion may explain the immunodeficiency features observed here despite the absence of childhood symptoms. Moreover, the X-MAID spectrum may include a range of linked conditions, from the classical SCID manifestations [1–5] to autoimmune phenotypes such as IBD-like disease [7] and, as reported by Kovács and colleagues, autoimmune thyroiditis and diabetes [14].

Currently, there is no international consensus or guidelines for treating X-MAID. Supportive management should depend on the severity and clinical features, and may include G-CSF, prophylactic antibiotics, and Ig-replacement therapy as needed [1, 2, 5], avoiding live vaccines during reimmunization [2], and considering allogeneic HSCT in severe cases [5].

## 4. Conclusions

Although repeated infections in early life are a common clinical presentation of X-MAID, we reported a case of adult-onset profound neutropenia and hemizygous MSN deletion with no history of significant childhood infections, cytopenias, or hypogammaglobulinemia. This report may be related to further variants of X-MAID and enrich the known spectrum of the disease.

## Figures and Tables

**Table 1:** Immune profiling at onset and 4-month follow-up visits.

Test	Onset	4-month follow-up	Reference range (units)
Absolute CD3 lymphocytes	1406	1123	622–2402 (cells/µL)
Absolute CD4 T cells	569	457	359–1519 (cells/µL)
Absolute CD8 T cells	756	572	109–897 (cells/µL)
Absolute CD19 lymphocytes	167	245	12–645 (cells/µL)
Absolute NK cells	151	96	24–406 (cells/µL)
CD4/CD8 ratio	**0.75 (L)**	**0.79 (L)**	0.92–3.72 (ratio)
Relative CD3 lymphocytes	81.4	75.9	57.5–86.2 (%)
Relative CD4 T cells	32.9	30.9	30.8–58.5 (%)
Relative CD8 T cells	**43.7 (H)**	**38.7 (H)**	12.0–35.5 (%)
Relative CD19 lymphocytes	9.6	16.6	3.3–25.4 (%)
Relative NK cells	8.7	6.5	1.4–19.4 (%)
IgG	1,318	1,210	700–1600 (mg/dL)
IgA	2.31	2.10	0.90–3.86 (g/L)
IgM	1.56	1.47	0.40–2.30 (g/L)
IgE	65.0	45.8	0–100 (IU/mL)

*Note:* Bold highlighting represents high (H) and low (L) values.

Abbreviations: Ig, immunoglobulin; NK, natural killer cells.

## Data Availability

The data supporting this study's findings are available from the corresponding author upon reasonable request.
